# Preliminary Assessment of Individual Zone of Optimal Functioning Model Applied to Music Performance Anxiety in College Piano Majors

**DOI:** 10.3389/fpsyg.2022.764147

**Published:** 2022-04-07

**Authors:** Zijin Yao, Yue Li

**Affiliations:** ^1^School of Arts, Beijing Language and Culture University, Beijing, China; ^2^Department of Music, Beijing Institute of Education, Beijing, China

**Keywords:** music performance anxiety, IZOF model, optimal performance, performance predicting, cognitive state anxiety (CA)

## Abstract

Individual zone of optimal functioning (IZOF) is a psychological model studied and applied to quantify athletes’ anxiety and predicts their achievement in sports competitions. This study aimed to determine the application of the IZOF model to evaluate music performance anxiety (MPA) in pianists because the causes of anxiety in athletes and musicians may be similar. A total of 30 college-level piano-major students were included in the study, and the anxiety level in performance was scored by the Competitive State Anxiety Inventory-2 questionnaire. In the first phase, participants recalled and self-scored the four important performances in the past year. Notably, seven piano teachers scored the performances. Both results were combined to identify the individual IZOF zone. Each student showed different anxiety scores for cognitive state anxiety (CA), somatic state anxiety (SA), and self-confidence (SC). In the second phase, all participants scored their anxiety level 1 day before the final performance, and the same judges evaluated the performance immediately afterward. A total of 60% of the participants who had at least two subscales inside the IZOF received performance scores greater than 90. In conclusion, the IZOF model provides information for both piano teachers and pianists to help review their anxiety intensity and predict their performance scores to some extent.

## Introduction

Research on music performance anxiety (MPA) has been conducted for several decades and is still ongoing (Fishbein et al., 1988; Steptoe, 2001; Kenny and Osborne, 2006; Kenny, 2011; Topoğlu, 2014; Guyon et al., 2020b). MPA is a globally negative and debilitating psychological phenomenon in musicians regardless of age, gender, experience, practicing time, and music genre (Brugués, 2011a,b; Studer et al., 2011; Barbar et al., 2014; Nusseck et al., 2015; Bannai et al., 2016; Sousa et al., 2016; van Fenema et al., 2017; Burin et al., 2019; Guyon et al., 2020a). MPA had been identified in music students and shown statistically significant differences in various psychological constructs, including optimism, self-efficacy, achievement motivation, and sensitivity to reward and punishment (Alzugaray et al., 2016). A significant relationship was reported between the age of starting musical training and the individual’s current perceived level of MPA; students who started at the age of 7 or younger showed lower levels of MPA (Zarza-Alzugaray et al., 2018). Furthermore, the MPA level increased among advanced conservatory students during their 4-year university-level studies (Casanova et al., 2018). A previous study revealed that 33.9% of participants had used substances to cope with MPA, and more than half of them had considered abandoning their musical studies. Participants who used substances had more frequent thoughts of giving up their musical career and had a higher level of MPA than control students (Orejudo Hernández et al., 2018). The relevance of family support for self-efficacy in public performance was mediated through MPA directly and showed consequent differences between genders (Zarza-Alzugaray et al., 2020). Social supports, such as the role of parents, teachers, and peers, were crucial for predicting self-efficacy for learning in students from advanced music schools (Orejudo et al., 2021). Nevertheless, MPA is a validated construct that can harm musicians’ performance quality and their careers (Osborne and Kenny, 2005; Yoshie et al., 2009; Davison, 2020).

Musicians may be ashamed to admit that they are suffering from performance anxiety (Bodner and Bensimon, 2008; Brugués, 2009). However, performance anxiety represented a series of psychosomatic manifestations and was a furtive concept for musicians, causing doubt about their performance quality (Lee, 1988). In addition, music educators had often consciously avoided this issue in their teaching process since anxiety management was typically beyond their training, talent, practice, experience, and dedication (Nideffer and Hessler, 1978).

Numerous strategies have been presented in previous studies and were shown to be widely used to control and improve the physical responses to MPA, including music-assisted progressive muscle relaxation, relaxation breathing training, yoga, physical activity, improvisation-assisted desensitization, psychoanalytic and cognitive-behavioral therapies, imagery-based interventions, acceptance and commitment therapy, music performance skills course, oxytocin intake, and expressive writing intervention (Kim, 2008; Su et al., 2010; Khalsa et al., 2013; Rocha et al., 2014; Studer et al., 2014; Spahn, 2015; Finch and Moscovitch, 2016; Kenny et al., 2016; Juncos et al., 2017; Cohen and Bodner, 2018; Fernholz et al., 2019; Zhukov, 2019; Clarke et al., 2020; Sabino et al., 2020; Shaw et al., 2020; Tang and Ryan, 2020).

Anxiety was thought to have both facilitated and attenuated individuals’ performances (Burton and Naylor, 1997). Performers with facilitative anxiety often described it as excitement, being pumped, or being “in the zone,” and they did not seek help from psychologists or other treatment professionals for assistance to reduce their anxiety (Lehrer et al., 1990; Robertson and Eisensmith, 2010). Wolfe also noted that MPA had positive effects on performance and explained these as an adaptive component of MPA (Wolfe, 1989). The adaptive component, also known as functional anxiety, readied the performer for the challenge ahead by directing preparatory arousal into practical task-oriented actions (Mor et al., 1995). Therefore, anxiety reduction may not be the most appropriate strategy for intervention to manage performance anxiety and achieve peak performance (Chamberlain and Hale, 2007). Increasing clinical reports, especially in the field of music performance, shows that some musicians need to experience pre-performance anxiety to perform at their best level (Nideffer and Hessler, 1978). In these cases, MPA was viewed as a more positive emotion in the performance of specific individuals (Kendrick et al., 1982; Brodsky, 1996; Kim, 2005; McGinnis and Milling, 2005).

Meanwhile, MPA was reported to be a more neutral concept and was viewed as a daily healthy aspect of stress and anxiety intrinsic to the music profession. Brodsky pointed out the complex designs of previous studies and revealed the misleading definitions and ineffective remedies for managing performance-related psychological problems in musicians, indicating that the interaction between anxiety level and actual performance remained in question and needed more research (Brodsky, 1996).

We would naturally hesitate to face these contradictory views and treatments relative to MPA. If there is a type of anxiety that facilitates performance, how would it present? If this anxiety feels differently to different individuals, what would be the difference between those who perceive anxiety as excitement and those who perceive anxiety as a catastrophe?

Various representative theories explained the relationship between performance and emotions (reflecting upon mental and physical arousal). Sports psychologists increasingly agreed that unidimensional approaches to the arousal-performance or anxiety-performance relationship were ineffective and simplistic (Hanin, 2000). Thus, approaches that used a single cumulative score of anxiety to demonstrate the relationship between performance and emotions were inadequate for examining an occupation with the complex emotional and motor skill requirements of music performance. More multidimensional approaches were called for anxiety-related research.

In the 1980s, Hanin introduced the theory of the individual zone of optimal functioning (IZOF), which proposed that an athlete’s performance was successful when his or her precompetitive anxiety was within or near the optimal zone (Hanin, 2000). It was a theoretical, multidimensional approach to describe, predict, and explain athletes’ performance-related, biopsychosocial states that affected individual activity. Athletes were asked to imagine their biopsychosocial states, and a personal IZOF was established to predict their future performances. The IZOF proposed that an athlete’s performance was successful when his or her pre-competitive anxiety was within or near the IZOF, which had been widely applied among athletes (Hanin, 2000, 2010; Harmison, 2006; Robazza et al., 2016, 2018; Ruiz et al., 2017, 2019; Cooper et al., 2021), and in physical activity at school (Robazza and Bortoli, 2005; Morano et al., 2020).

Compared to research exploring emotions in sports and the individual optimal zone, far less research had been published applying IZOF theory on MPA treatments (Yao, 2016). In music performance circumstances, as the subjective experience of anxiety varied from person to person, the optimal zone differed from person to person. By defining the optimal functioning zone for individual pianists and predicting upcoming performance results, this study verified that IZOF can still effectively describe, predict, explain, and regulate piano performance-related bio-psycho-social states as well. In particular, the location and width of the IZOF helped determine a possible range of performance scores.

We conducted a pilot study and found the IZOF zone in two cases. The best performances by these two pianists were presented in the IZOF zone with a significantly higher IZOF score than the average out-of-zone score (Yao, 2016). In this retrospective study, IZOF was assumed to be fully applied in piano performance analysis. Moreover, the performance prediction process showed that it was vital to know each pianist’s IZOF since it varied widely from person to person and may determine each pianist’s personal mental and physical practice.

This study aimed to clarify pianists’ personal IZOF zone, assess the contribution of MPA on their optimal performance, and examine the prediction accuracy of future performance results. This information may help pianists to prepare more and regulate their mental and physical states before future performances.

## Materials and Methods

### Participants

**FIGURE 1:**
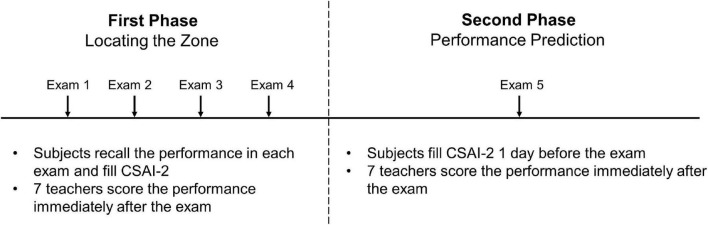
The study protocol.

A total of 30 participants aged between 18 and 24 years were enrolled, including 7 male and 23 advanced female pianists. Participants were all undergraduates with piano majors in a conservatory in Beijing, China. The students came from 13 different provinces in China. At the time of the survey, 6 were sophomores, 10 were juniors, and 14 were seniors, all in a 4-year bachelor’s degree system. The study protocol is shown in Figure 1.

### Ethics Statement

This study was conducted anonymously. No names or other identifying personal data were recorded. Consent forms were sent to the participants to be filled out and signed before the study began, and all included students provided signed informed consent. This study had no risks associated with the physical or psychological state of the participants.

### Scoring the Piano Performances

The performances of each participant were evaluated by a group of seven professional college piano teachers. Each teacher evaluated the performance of each participant on a scale of 1–100, where 1 = worst possible performance and 100 = best possible performance. The judges were told to score the performance immediately after the performances based on the participants’ playing. The score was to represent an overall impression of their performances. The highest and lowest scores were removed in the final grading, and the remaining five scores were selected and used to calculate the average score of the final performance result.

### The First Phase of This Study: Locating the Zone

In the first phase of the study, participants were required to reflect upon their past four performances (2 mid-term and 2 final examinations in the past academic year) and complete the Competitive State Anxiety Inventory-2 (CSAI-2). The CSAI-2 was a self-reported inventory with 27 simple questions. It took about 5 min to complete each evaluation and was used to measure the performance anxiety state. It showed the anxiety level of the three different dimensions (subscales), namely, somatic anxiety (SA), cognitive anxiety (CA), and self-confidence (SC). The subscale scores of each dimension ranged from 9 to 36. According to data collected from the CSAI-2, the IZOF in SA, CA, and SC dimensions was identified separately for each pianist. Statistically, IZOF is shown as M ± 1/2 SD. M is the mean of CSAI-2 subscales corresponding to the personal best piano performance, and SD is the standard deviation of CSAI-2 subscales. The difference (D) between the mean of in-IZOF and out-of-IZOF was calculated as performance scores and the percentage of the CSAI-2 subscale when the participant was below, in, or above the IZOF zone. Furthermore, the relative efficacy of the method for determining anxiety was compared by the percentage of outstanding or less-than-outstanding performance on the IZOF (i.e., percentage of outstanding performances inside the IZOF and less-than-outstanding outside the IZOF). We identified the outstanding performance as mean plus one SD of the 30 pianists with four performance scores. Outstanding performances in the first phase of the study were set at 92 (performance score mean: 88.98, SD: 3.47). Performance evaluations were made by seven piano teachers of judges as mentioned earlier.

### The Secondary Phase of This Study: Performance Prediction

In the second phase, we evaluated the predictive accuracy of CSAI-2. The IZOF zone was identified again. Predictions can be projected for each subject based on their answers to the CSAI-2 before an upcoming jury. The IZOF theory was used for performance prediction and analysis. Subjects answered the CSAI-2 on the day before their final jury of the semester. Anxiety intensity from three subscales was compared with the upper and lower thresholds of corresponding zones to see if the subjects’ performances fell within their zones. After the final jury, performance evaluations were made and collected by the same group of judges using the same method. Data were collected to examine the hypothesis that the IZOF model can help to predict the upcoming performance and be fully applied within the piano performance anxiety description, explanation, assessment, and performance prediction.

### Statistical Analysis

Continuous data were presented as mean, SD, minima, and maxima. Categorical data were presented as count and percentage. In the first phase of this study, we conducted the IZOF for each pianist and calculated the performance score difference between in-IZOF and out-of-IZOF. Furthermore, we calculated the percentage of outstanding or less-than-outstanding performance on the IZOF. In the second phase of this study, a correlation between how many pianists were in or out of their IZOF and performance was analyzed. We calculated the predictive in-zone performances and the statistical description of the actual performance score. Scatterplots were drawn to show the distance between pre-performance CSAI-2, their IZOF, and the jury’s judged performance score. The distance from the closest zone border and performance score was conducted, and the distance was set to 0 if a value falls within the zone. We used the Spearman correlation coefficient to show the correlation between the distance and the performance score because these data did not show normal distribution. A two-sided *p*-value of <0.05 was regarded as statistically significant. Data management and statistical analyses were conducted using SAS version 9.4 software (SAS Institute, Inc.).

## Results

Table 1 summarizes the overall descriptive statistics for CSAI-2 subscales. The average CA score of 30 students corresponding to the best performances is 18.0 ± 4.9 (minimum-maximum: 11–34), the average SA score is 17.3 ± 4.8 (minimum-maximum: 11–26), and the average SC score is 20.9 ± 5.5 (minimum-maximum: 11–31). Students’ states of CA, SA, and SC are different according to personal best performance. The individual IZOF by M ± 1/2 SD is shown in Supplementary Table 1.

**TABLE 1:** Overall descriptive statistics for CSAI-2 Subscales.

	S	Cognitive anxiety		Somatic anxiety		Self-confidence	
				
		M	1/2 SD	M	1/2 SD	M	1/2 SD
Mean	92.8	18.0	1.3	17.3	1.2	20.9	1.6
Std	1.7	4.9	0.8	4.8	0.8	5.5	0.8
Median	92	17	1.1	16	1.1	21	1.3
Min	90	11	0.3	11	0.3	11	0.6
Max	97	34	4.3	26	4.1	31	4.1

*IZOF, individual zone of optimal functioning; M, mean of CSAI-2 subscales corresponding to the best performance; SD, standard deviation of CSAI-2 subscales; S, score of performance.*

The D (mean of in-IZOF–mean of out-of-IZOF) ranks from large to small showed that the differences in SA and SC have a similar trend (Supplementary Table 1). The different levels of performance scores in CA, SA, and SC range from 3.3 to 11. The average D in CA, SA, and SC are 6.2 ± 1.9, 6.2 ± 2.0, and 6.1 ± 2.1, respectively. For example, if we consider student #14, the different levels in all three subscales reach 11 and three performance scores are in the IZOF area (75%) with the best performance score of 91 ± 1 points.

Table 2 shows the average correct classification (in percentage) of outstanding performances inside IZOF and less-than-outstanding performances outside IZOF with the CSAI-2 questionnaires. Outstanding performances were set at a score of 92 points. The IZOF in CA, SA, and SC results in an average of 84.2, 80.8, and 77.5%, respectively, correct predictions (all range of the three subscales: 50–100%).

**TABLE 2:** Average correct classification (in percentage) of outstanding performances* inside IZOF and less-than outstanding performances outside IZOF with the CSAI-2 questionnaires.

CSAI-2 Subscales	Mean	SD	Min	Max
Cognitive anxiety	84.2	18.0	50.0	100.0
Somatic anxiety	80.8	19.3	50.0	100.0
Self-confidence	77.5	19.0	50.0	100.0

*Outstanding performances was set at 92 (performance score mean: 8 8.98, SD: 3.47).

**FIGURE 2:**
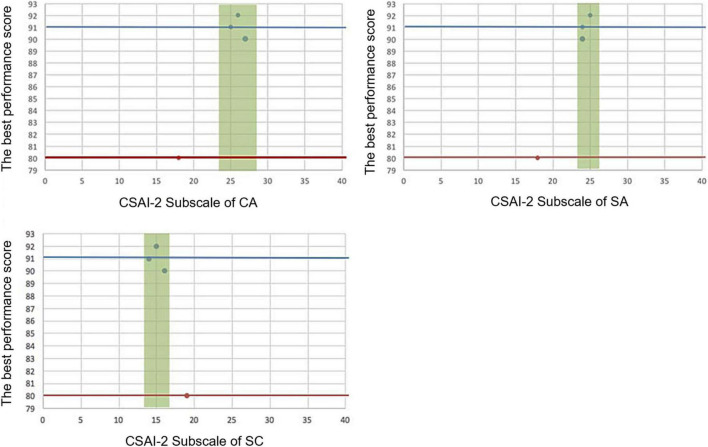
The best performance scores and CSAI-2 subscales for participant #14. The green band represents the IZOF of participant #14. The red and blue horizontal lines represent the lowest and highest scores of the best performance scores. The blue spots indicate the performance scores.

**FIGURE 3:**
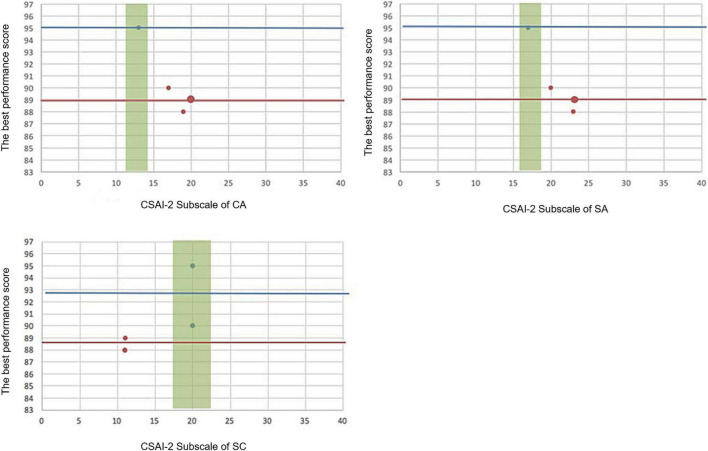
The best performance scores and CSAI-2 subscales inside or outside the IZOF for participant #29. The green band represents the IZOF of participant #29. The red and blue horizontal lines represent the lowest and highest scores of the best performance scores. The blue spots indicate the performance scores.

Two figures were presented to display two contrasting cases of personal IZOF. Figure 2 shows the performance distribution in the three subscales of student #14. It shows that if the student has a high CA (IZOF: 24.0–28.0) and SA (IZOF: 23.4–26.6) and a low SC (IZOF: 13.9–16.1), he or she would perform well. Figure 3 shows that if student #29 has a low CA (IZOF: 18.0–20.0) and SA (IZOF: 15.6–16.4) and a high SC (IZOF: 20.8–25.2), he or she would have a less-than-ideal performance.

In the second phase study, the 30 participants conducted the new IZOF. Table 3 shows the prediction in-zone performances and the statistical description of the actual performance score. A total of 14 (46.7%) students have all three subscales inside the IZOF, and the average performance score is 93.4 ± 1.5 (minimum-maximum: 91–96). A total of 60% of the participants have at least two subscales inside the IZOF and also receive performance scores ≥90 out of 100. In total, 10 (33.3%) students have none of the subscales inside the IZOF, and the average performance score is 86.2 ± 1.4 (minimum-maximum: 84–88). Totally, 18 (60%) and 16 (53.3%) students have CA and SA scores that fall above or in the IZOF, respectively, while 12 (40%) students have SC scores that fell below the IZOF.

**TABLE 3:** Predictive in-zone performances and the statistic description of actual performance score.

Combination	SA	CA	SC	N	%	Mean	SD	Median	Min	Max
1	In	In	In	14	46.7%	93.4	1.5	93.5	91	96
2	In	In	Out	1	3.3%	95	–	95	–	–
3	In	Out	In	2	6.7%	92	1.4	92	91	93
4	Out	In	In	1	3.3%	90	–	90	–	–
5	In	Out	Out	1	3.3%	96	-	96	–	–
6	Out	In	Out	–	–	–	–	–	–	–
7	Out	Out	In	1	3.3%	88	–	88	–	–
8	Out	Out	Out	10	33.3%	86.2	1.4	86	84	88

**FIGURE 4:**
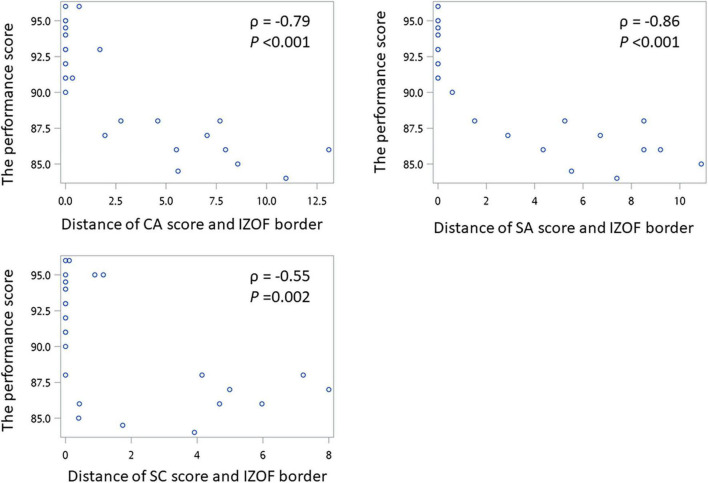
The scatterplot of distance from closest zone border and performance score.

Figure 4 shows the correlation between distance from closest zone border and performance score. The distance of CA, SA, and IZOF has significant strong negative correlation with the pianist’s performance score (CA: ρ = −0.79, *p* < 0.001; SA: ρ = −0.86, *p* < 0.001). The distance of SC and IZOF has a moderate negative correlation with the pianist’s performance score (CA: ρ = −0.55, *p* = 0.002).

## Discussion

This is the first study to apply IZOFs to musicians using CSAI-2 subscales. The results have verified the individual nature of each pianist relative to each subscale and demonstrated the zone’s efficiency in describing the relationship between MPA and optimal performance. The IZOF theory can be used for pre-performance anxiety analysis and performance prediction.

The IZOF zone was found for two cases in our previous pilot study. All of their best performances were presented in the IZOFs, and their average in-zone performance score was significantly better than the average out-zone score (Yao, 2016). This study further revealed the regulation and relationship between an individual’s anxiety intensity and their piano performance results. Everyone has different optimal levels of anxiety intensity. Therefore, applying the IZOF theory to music performance offers a new perspective on managing performance anxiety. With the help of the IZOF model, the study can define the “zone” in a quantified and measurable way. Moreover, with more than four sets of CSAI-2 data provided by pianists, the IZOF model may well be applied to predict pianists’ upcoming performances more precisely.

Music performance anxiety has been observed from different perspectives and studied with countless methods for many years, and researchers will continue studying this area with the help of cognitive and psychological science as it is developed. However, no matter how deeply this area has been studied, individual differences in reaction to performance anxiety issues cannot be denied, especially those of advanced music majors in colleges. Music interpretation is based on technique but is an emotion-supported performance activity. It involves a great deal of personal and emotional investment, which increases uncertainty and contributes to anxiety. Individual reactions to MPA vary widely among college-level pianists. In China, students enrolled in music conservatories have already achieved an advanced level of proficiency in piano performance. However, not all of them are aware of their optimal zone for performance and attempt to master every public performance consistently with applied consciousness. As a result, even after years of training, only a few piano majors end up with a career in professional performance. With the application of the IZOF model, young pianists may become aware of certain dimensions that impact their performances in addition to technical skills and finger abilities. Therefore, knowing that the IZOF may help to enhance performance and improve personal satisfaction maybe even more important than deciding whether one should continue a performance career despite their MPA issues.

One participant who has a performance score greater than 90 has only one subscale inside the IZOF (Table 3, Combination 5). The participant’s CA score is 19 and SC score is 28, both are very close to the lower thresholds of the optimal CA zone (19.68) and the lower thresholds of the optimal SC zone (28.11). This contradictory result may be eliminated by improving the accuracy of identifying the IZOF zone. Increasing the measured frequency of conducting IZOF or using prospective studies instead of recalling may help with improvement.

### Limitations

This study has several limitations. First, although scholars in sports psychology have called for testing the IZOF model in more performance-related domains (Spielberger, 2013), few studies of MPA have adopted it as an applicable theory. Therefore, only limited resources can be found for comparison. Second, MPA might not be the only component affecting piano performance. The effects of other factors such as self-efficacy or social support may be underestimated and need to be considered. Third, fewer piano juries and participants may result in biased analyses and restrictions associated with future performance prediction. Piano juries are typically held four times per year, far less frequently than sports performances. Fourth, subjects were told to reflect on their most impressive performance to fulfill the retrospective recollection, which may increase the difficulty of defining the precise zone based upon fewer recollections and may result in inherent biases. Fifth, the lack of long-term data for tracking may decrease the accuracy of defining the IZOFs. Finally, since the scores did not show fluctuation for well-trained advanced performing musicians, the differences in performance scores for each person were very subtle (e.g., the lowest score was 84 and the highest was 95 on a scale of 0–100), which may affect the accuracy of prediction.

## Conclusion

Personal IZOF zones were identified for each of the 30 pianists. Notably, 60% of the participants had at least two subscales within the IZOF and also received performance scores ≥90 out of 100. The IZOF model provides information for both piano teachers and pianists to help review their anxiety intensity and predict their performance scores to some extent.

## Data Availability Statement

The original contributions presented in the study are included in the article/Supplementary Material, further inquiries can be directed to the corresponding author.

## Author Contributions

ZY: guarantor of integrity of the entire study, study concepts, study design, definition of intellectual content, literature research, data analysis, statistical analysis, manuscript preparation, manuscript editing, and manuscript review. YL: literature research, clinical studies, experimental studies, and data acquisition. Both authors contributed to the article and approved the submitted version.

## Conflict of Interest

The authors declare that the research was conducted in the absence of any commercial or financial relationships that could be construed as a potential conflict of interest.

## Publisher’s Note

All claims expressed in this article are solely those of the authors and do not necessarily represent those of their affiliated organizations, or those of the publisher, the editors and the reviewers. Any product that may be evaluated in this article, or claim that may be made by its manufacturer, is not guaranteed or endorsed by the publisher.
