# Supplementary material for: Preliminary Assessment of Individual Zone of Optimal Functioning Model Applied to Music Performance Anxiety in College Piano Majors
Source: Front Psychol. 2022 Apr 7;13:764147. doi: 10.3389/fpsyg.2022.764147 (PMC9021823; doi:10.3389/fpsyg.2022.764147)
Supplement: Supplementary file 1 [file Table_1.docx]

**Supplementary Table 1. In-of-IZOF and Out-of-IZOF Performance Differences for CSAI-2 Subscales.**

| **ID no.** | **Cognitive Anxiety** | | |  | **Somatic Anxiety** | | |  | **Self-Confidence** | | |  |
| --- | --- | --- | --- | --- | --- | --- | --- | --- | --- | --- | --- | --- |
|  | **In-of-zone** | **Out-of-zone** | **D** |  | **In-of-zone** | **Out-of-zone** | **D** |  | **In-of-zone** | **Out-of-zone** | **D** |  |
| #14 | 3, 91±1 | 1, 80 | 11 |  | 3, 91±1 | 1, 80 | 11 |  | 3, 91±1 | 1, 80 | 11 |  |
| #8 | 2, 91.8±0.4 | 2, 82.5±0.7 | 9.3 |  | 2, 90±1 | 2, 84.5±0.8 | 9.3 |  | 2, 91.8±0.4 | 2, 82.5±0.7 | 9.3 |  |
| #21 | 2, 92.5±0.7 | 2, 83.5±3.5 | 9 |  | 2, 92.5±0.7 | 2, 83.5±3.5 | 9 |  | 3, 90.3±3.8 | 1, 81 | 9.3 |  |
| #10 | 2, 91±0 | 2, 82±1.4 | 9 |  | 1, 84 | 3, 88±2.8 | 9 |  | 2, 91±0 | 2, 82±1.4 | 9 |  |
| #1 | 1, 94 | 3, 85±1.7 | 9 |  | 1, 94 | 3, 85±1.7 | 9 |  | 1, 94 | 3, 85±1.7 | 9 |  |
| #25 | 2, 92.5±0.7 | 2, 85±1.4 | 7.5 |  | 2, 92.5±0.7 | 2, 85±1.4 | 7.5 |  | 2, 92.5±0.7 | 2, 85±1.4 | 7.5 |  |
| #20 | 1, 94 | 3, 86.7±3.8 | 7.3 |  | 2, 92.5±2.1 | 2, 84.5±0.7 | 8 |  | 2, 92.5±2.1 | 2, 84.5±0.7 | 8 |  |
| #16 | 1, 92 | 3, 84.7±1.5 | 7.3 |  | 1, 92 | 3, 84.7±1.5 | 7.3 |  | 1, 92 | 3, 84.7±1.5 | 7.3 |  |
| #11 | 1, 91 | 3, 83.7±2.1 | 7.3 |  | 2, 91.5±0.9 | 2, 87±1.6 | 7.3 |  | 2, 88.5±3.5 | 2, 82.5±0.7 | 6 |  |
| #9 | 2, 92±1.4 | 2, 85±1.4 | 7 |  | 1, 86 | 3, 85±1.9 | 7 |  | 2, 92±1.4 | 2, 85±1.4 | 7 |  |
| #28 | 2, 93.5±0.7 | 2, 86.5±7.8 | 7 |  | 2, 93±1.4 | 2, 87±8.5 | 6 |  | 2, 93.5±0.7 | 2, 86.5±7.8 | 7 |  |
| #27 | 2, 91±1.4 | 2, 84±2.8 | 7 |  | 2, 91±1.4 | 2, 84±2.8 | 7 |  | 2, 91±1.4 | 2, 84±2.8 | 7 |  |
| #17 | 1, 94 | 3, 87.7±1.5 | 6.3 |  | 1, 94 | 3, 87.7±1.5 | 6.3 |  | 1, 94 | 3, 87.7±1.5 | 6.3 |  |
| #29 | 1, 95 | 3, 89±1 | 6 |  | 1, 95 | 3, 89±1 | 6 |  | 2, 92.5±3.5 | 2, 88.5±0.7 | 4 |  |
| #22 | 2, 95±2.8 | 2, 89±0 | 6 |  | 2, 95±2.8 | 2, 89±0 | 6 |  | 1, 97 | 3, 90.3±2.3 | 6.7 |  |
| #13 | 1, 90 | 3, 84.3±4.2 | 5.7 |  | 1, 82 | 3, 85±1.10 | 7.5 |  | 2, 89.5±0.7 | 2, 82±1.4 | 7.5 |  |
| #4 | 2, 90±0 | 2, 84.5±0.7 | 5.5 |  | 2, 90±0 | 2, 84.5±0.7 | 5.5 |  | 2, 90±0 | 2, 84.5±0.7 | 5.5 |  |
| #7 | 1, 94 | 3, 88.7±1.5 | 5.3 |  | 2, 91.5±0.8 | 2, 87±1.5 | 5.3 |  | 2, 91.5±3.5 | 2, 88.5±2.1 | 3 |  |
| #6 | 1, 92 | 3, 87±1 | 5 |  | 1, 88 | 3, 88±2.7 | 5 |  | 1, 92 | 3, 87±1 | 5 |  |
| #30 | 2, 92.5±0.7 | 2, 87.5±2.1 | 5 |  | 2, 92.5±0.7 | 2, 87.5±2.1 | 5 |  | 2, 92.5±0.7 | 2, 87.5±2.1 | 5 |  |
| #26 | 2, 95±1.4 | 2, 90±0 | 5 |  | 1, 96 | 3, 91.3±2.3 | 4.7 |  | 2, 95±1.4 | 2, 90±0 | 5 |  |
| #2 | 2, 91.5±0.7 | 2, 86.5±0.7 | 5 |  | 1, 92 | 3, 88±2.6 | 4 |  | 1, 92 | 3, 88±2.6 | 4 |  |
| #24 | 2, 94.5±0.7 | 2, 89.8±0.4 | 4.8 |  | 2, 94.5±0.7 | 2, 89.8±0.4 | 4.8 |  | 2, 94.5±0.7 | 2, 89.8±0.4 | 4.8 |  |
| #5 | 1, 92 | 3, 87.3±0.6 | 4.7 |  | 1, 90 | 3, 85±1.8 | 4.7 |  | 2, 90±2.8 | 2, 87±0 | 3 |  |
| #3 | 2, 91.5±0.7 | 2, 87±1.4 | 4.5 |  | 2, 91.5±0.7 | 2, 87±1.4 | 4.5 |  | 3, 90.3±2.1 | 1, 86 | 4.3 |  |
| #19 | 2, 91.5±0 | 2, 87±1.4 | 4.5 |  | 2, 91.5±0 | 2, 87±1.4 | 4.5 |  | 2, 91.5±0 | 2, 87±1.4 | 4.5 |  |
| #18 | 2, 91.5±0.7 | 2, 87±1.4 | 4.5 |  | 2, 91.5±0.7 | 2, 87±1.4 | 4.5 |  | 2, 91.5±0.7 | 2, 87±1.4 | 4.5 |  |
| #12 | 3, 93.3±0.6 | 1, 89 | 4.3 |  | 2, 90±2 | 2, 84.5±0.9 | 2.3 |  | 3, 93.3±0.6 | 1, 89 | 4.3 |  |
| #15 | 2, 91.5±0.7 | 2, 88±0 | 3.5 |  | 2, 91.5±0.7 | 2, 88±0 | 3.5 |  | 2, 91.5±0.7 | 2, 88±0 | 3.5 |  |
| #23 | 1, 91 | 3, 87.7±0.6 | 3.3 |  | 1, 91 | 3, 87.7±0.6 | 3.3 |  | 1, 91 | 3, 87.7±0.6 | 3.3 |  |
| average | - | - | 6.2±1.9 | | - | - | 6.2±2.0 | | - | - | 6.1±2.1 | |

Data are presented as n, mean ± SD.

IZOF: Individual Zones of Optimal Functioning; D: mean of In-of-IZOF - mean of Out-of-IZOF; SD: Standard Deviation of the performance score.
